# Tenofovir vs. Entecavir on Outcomes of Hepatitis B Virus-Related Hepatocellular Carcinoma after Radiofrequency Ablation

**DOI:** 10.3390/v14040656

**Published:** 2022-03-22

**Authors:** Zili Hu, Huilan Zeng, Jingyu Hou, Juncheng Wang, Li Xu, Yaojun Zhang, Minshan Chen, Zhongguo Zhou

**Affiliations:** 1 Department of Liver Surgery, Sun Yat-sen University Cancer Center, Guangzhou 510060, China; huzl1@sysucc.org.cn (Z.H.); cenghl@sysucc.org.cn (H.Z.); houjy@sysucc.org.cn (J.H.); wangjch@sysucc.org.cn (J.W.); xuli@sysucc.org.cn (L.X.); zhangyuj@sysucc.org.cn (Y.Z.); 2 Collaborative Innovation Center for Cancer Medicine, State Key Laboratory of Oncology in South China, Sun Yat-sen University Cancer Center, Guangzhou 510060, China

**Keywords:** hepatocellular carcinoma, chronic hepatitis B, entecavir, tenofovir disoproxil fumarate, comparative effectiveness

## Abstract

For patients with hepatitis B virus (HBV)-related hepatocellular carcinoma (HCC) treated with curative radiofrequency ablation (RFA), the effect of entecavir (ETV) vs. tenofovir disoproxil fumarate (TDF) on recurrence-free survival (RFS) and overall survival (OS) remains unclear. We aimed to compare the outcomes of patients receiving ETV or TDF after RFA. This study consecutively collected patients who were treated with ETV (*n* = 202) or TDF (*n* = 102) for chronic hepatitis B (CHB) after curative RFA of HCC from December 2015 to January 2021 at Sun Yat-sen University Cancer Center. There were 130 patients in the ETV group and 77 patients in the TDF group after we performed 1-to-n propensity score matching. Kaplan–Meier and Cox regression analyses were performed to validate possible risk factors for RFS and OS. In addition, we estimated the curative effect of ETV and TDF for HBV-related hepatitis by recording the change in serum HBV DNA and ALBI grade after RFA. During the study period (median 34.1 (interquartile range: 19.6–47.4 months) months), 123 (40.5%) patients suffered HCC recurrence, and 15 (4.9%) died. In the full cohort, the probability of HCC recurrence (41.6% vs. 37.3%, *p* = 0.49) and overall survival (95% vs. 95.1%, *p* = 0.39) at 5 years were similar between the ETV and TDF groups. In the matched cohort, HCC recurrence (40.8% vs. 40.3%, *p* = 0.35) and overall survival (96.9% vs. 93.5%, *p* = 0.12) at 5 years were similar between the ETV and TDF groups. Furthermore, the early RFS (<2 years) did not differ significantly between the two groups in the full and matched cohorts (*p* = 0.26, *p* = 0.13). Compared with the ALBI grade before RFA, the ALBI grade of 80 patients (41%) remained stable or improved in the ETV group and 64 patients (64%) in the TDF group (*p* < 0.001). The mean time of serum HBV DNA reduction to 0 was 9.13 (95% CI: 5.92–12.33) and 2.75 (95% CI: 2.01–3.49) months in the ETV and TDF groups, respectively (*p* = 0.015). The RFS and OS of patients after curative RFA for HCC were not significantly different between the ETV and TDF groups. TDF therapy was associated with a better effect of protecting liver function and reducing the load of HBV. Further validation studies are needed.

## 1. Introduction

Hepatocellular carcinoma (HCC) is the fifth most common cancer and the second largest cause of cancer-related mortality in the world [1,2,3]. Hepatitis B virus (HBV) is a major cause of HCC, especially in the Asia-Pacific region, including China [4,5]. Recent studies show that hepatitis viral load is an independent risk factor for the prognosis of HCC after hepatectomy or RFA in patients with chronic hepatitis B (CHB) [6,7,8,9,10]. Reducing HBV load through treatment with nucleos(t)ide analogs (NAs) may lower the risk of HCC recurrence and improve the survival of patients after hepatectomy and RFA [11,12,13,14]. Therefore, antiviral therapy is essential for patients after curative therapy for HCC.

Hepatectomy and radiofrequency ablation (RFA) are the first-line curative therapeutics for early-stage HCC. Several randomized controlled trials (RCTs) have shown that the long-term prognosis is similar between hepatectomy and RFA in early-stage HCC patients [15,16,17,18,19,20]. In addition, RFA has the advantage of being less invasive than surgical resection [15]. RFA is recommended as a curative therapeutic for patients with early-stage HCC according to guidelines of the European Society for Medical Oncology (ESMO) [21] and the American Association for the Study of Liver Disease (AASLD) [22]. RFA may be a better choice for patients with early-stage HCC. Consequently, we focus on the patients after RFA.

Currently, entecavir (ETV) and tenofovir (TDF) are equally recommended as first-line NA treatments for patients with CHB because of their high antiviral effects and high genetic barrier to drug resistance [23,24,25]. The effect of ETV vs. TDF therapy on prognosis after hepatectomy remains controversial. Recently, a study showed that TDF treatment was associated with a significantly lower rate of HCC recurrence and better overall patient survival than ETV therapy among patients who underwent curative hepatectomy for HBV-related HCC [26]. In contrast, another study showed that the rate of HCC recurrence and overall patient survival were not significantly different between the ETV and TDF groups among patients after curative therapy for HBV-related HCC [27]. However, until now, there has been no study comparing the effect of ETV vs. TDF on the prognosis of patients with HBV-related HCC treated with curative RFA.

Therefore, in this study, we aimed to compare long-term prognosis, including tumor recurrence and overall survival, and short-term effects between an entecavir treatment group and a tenofovir treatment group with HBV-related HCC after curative RFA.

## 2. Materials and Methods

### 2.1. Study Design and Subjects

This is a historical cohort study of patients who were treated with either TDF or ETV for CHB after curative RFA for HCC from December 2015 to January 2021. The source population was obtained from a historical cohort of 830 consecutive patients at Sun Yat-sen University Cancer Center (SYSUCC). Of these, 304 patients who were treated with either ETV (*n* = 202) or TDF (*n* = 102) were included in the analysis as the study population. All study patients had HCC of BCLC A-B.

**Figure 1:**
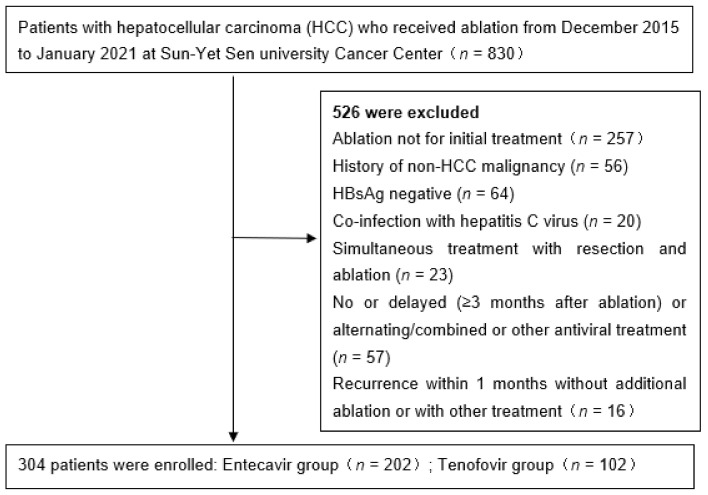
Flow diagram of enrolled patients.

We excluded patients who met any of the following criteria: any previous treatment for HCC via other modalities; history of non-HCC malignancy, coinfection with hepatitis C virus, simultaneous treatment with resection and radiofrequency ablation, incomplete ablation for HCC at magnetic resonance imaging (MRI) obtained 1 month after RFA at reexamination, lack of follow-up within 6 months, and previous treatment with antiviral drugs other than ETV or TDF or alternating/combined use of NAs. To exclude the potential influence of technical factors for the RFA procedure on the patient’s outcome, patients with local tumor progression (LTP) during follow-up (*n* = 33) were further excluded (Figure 1).

The diagnosis of HCC was made on the basis of American Association for the Study of Liver Diseases (AASLD) guidelines [22]. Chronic hepatitis B was defined as HBsAg-positive for at least 6 months. The indication for the initiation of antiviral treatment was based on the guidelines of prevention and treatment for chronic hepatitis B (2019 version): HBsAg-positive HCC patients are recommended to accept NA therapy [28]. Cirrhosis was also defined clinically by findings of ultrasonography (US) [29]. This study was approved by the ethics committee of Sun Yat-sen University Cancer Center (Protocol code: B2019-008-01, date: March 2019).

### 2.2. RFA Procedure

All patients underwent contrast-enhanced ultrasound before RFA, and we confirmed the lesion again using ultrasound on the same day before RFA. The patients were given intravenous anesthesia and local anesthesia during RFA. All procedures were performed by one (Chen MS) ablation expert with 20 years of experience under real-time ultrasound guidance based on a previous study [30]. The ZW-II RFA system (Dalong South Technical Co., Ltd., Shenzhen, China) was used for ablation. The electrode with an exposed tip was percutaneously inserted to the bottom of the tumor, avoiding vital structures (such as large vessels and biliary ducts). RFA was initiated with 30 W of power, and the power was increased 10 W per minute to 90 W. RF was applied until either tissue impedance increased sharply or 15 min had elapsed. Then, we scanned the residual lesion to confirm whether the ablation area had covered the entire tumor; otherwise, a second ablation was performed to achieve a satisfactory ablation area. Finally, we ablated the needle to prevent bleeding and needle track seeding. After RFA, patients were sent back to the ward with vital signs monitored.

### 2.3. Outcomes and Follow-Up after RFA

The primary outcome of this study was recurrence-free survival (RFS) and overall survival (OS), and the secondary outcome was the short-term effect of ETV and TDF on HBV-related hepatitis. All patients underwent enhanced MRI and laboratory tests, including serum alpha-fetoprotein (AFP) level, HBV DNA, and liver function tests, at the first month after RFA, every 3 months for the first 2 years after RFA, and every 6 months thereafter. Tumor recurrence after initial RFA was classified into three subtypes according to the reporting guidelines [31]: LTP, intrahepatic distant recurrence (IDR), and extrahepatic recurrence (ER). We defined IDR and ER as tumor recurrence, whereas LTP was excluded because our aim was to investigate the role of antiviral treatment on tumor recurrence after RFA. If recurrence was identified during the follow-up period, patients were treated with RFA, surgery, radiation therapy, hepatic arterial chemoembolization, or systemic therapy (targeted therapy and/or immunotherapy) based on the discussion of a multidisciplinary team (MDT) for HCC treatment according to the characteristics of the recurrent tumor, liver function, and the general condition of the patient.

### 2.4. Statistical Analysis

Categorical variables are described as frequencies and percentages. Continuous variables are described as the mean ± standard deviation (SD) and median with interquartile range for parametric and nonparametric variables, respectively. In the full cohort, 202 patients received ETV therapy, and 102 patients received TDF therapy after RFA. To minimize the difference between the two groups, we performed 1-to-n propensity score matching in consideration of variables described in Table 1 (age, sex, diabetes, hypertension, complications, cirrhosis, tumor size, tumor number, platelet count, PT, APTT, albumin, total bilirubin, ALT, AST, log_10_HBV DNA, AFP, Child–Pugh class, ALBI grade). The caliper width was 0.2 of the standard deviation of the logit of the propensity score. The propensity score matching was conducted by R package “MatchIt”. After matching, there were 130 patients in the ETV group and 77 patients in the TDF group. The baseline characteristics of the matched cohort are described in Table 2, which were balanced between the two groups. Kaplan–Meier curves were used to estimate RFS, OS, and the time of serum HBV DNA clearance of the two patient groups and were compared by the log-rank test in full and matched cohorts. We defined tumor recurrence, death, and serum HBV DNA reduced to 0 as the events for RFS, OS, and time of serum HBV DNA clearance, respectively. The follow-up duration was defined as the interval between the first RFA and either the incidence of event or the last follow-up time (1 July 2021). We used univariable and multivariable Cox proportional hazards models to assess the risk factors for tumor recurrence and overall survival after RFA in the full cohort. We selected variables that were statistically significant in the univariable analysis (*p* < 0.05) for multivariable analysis, which was performed using a forward conditional stepwise procedure to avoid multicollinearity. All statistical analyses were conducted using R statistical software (version 3.6.3; R Foundation for Statistical Computing, Vienna, Austria, https://www.R-project.org/, accessed on 10 March 2020) and SAS (version 26.0, SAS Institute, Cary, NC, USA). The R package MatchIt was used to construct the matched cohort.

## 3. Results

### 3.1. Baseline Characteristics

In this study, 304 patients were included, of which 202 patients were treated with ETV and 102 patients were treated with TDF after RFA. The baseline characteristics of the two groups are described in Table 1. The median duration of follow-up for all patients was 34.1 months (interquartile range: 19.6–47.4 months). There was no significant difference in age, sex, basic disease, tumor size, tumor number, platelet count, PT, APTT, ALB, TBIL, HBV DNA, AFP, Child–Pugh class status, or ALBI grade between the two groups. However, the levels of ALT and AST in the TDF group were significantly higher than those in the ETV group (*p* = 0.014, 0.015). Additionally, more patients suffered complications in the ETV group (*p* = 0.004) than in the TDF group after RFA. The main complications were fever and abdominal pain. All patients with complications were relieved after symptomatic treatment. The number of patients with cirrhosis was significantly higher in the ETV group than in the TDF group (87% vs. 74%, *p* = 0.008).

To minimize the effect of potential confounders in the comparison of HCC RFS and OS between the ETV and TDF groups, we generated a matched patient cohort by propensity score matching. Baseline characteristics between the two groups after propensity score matching are described in Table 2. After matching, 130 patients were selected in the ETV group and 77 in the TDF group. There was no significant difference in the clinical variables described in Table 1 between the two groups, and it was considered that covariate balance was achieved (Table 2).

### 3.2. HCC Recurrence

**Figure 2:**
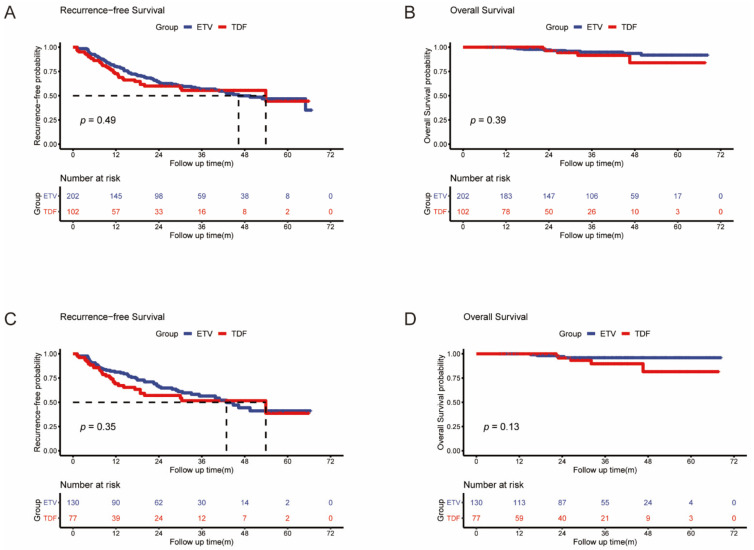
Kaplan–Meier curves of HCC recurrence (**A**) and overall survival (**B**) after RFA between the two groups in the full cohort; Kaplan–Meier curves of HCC recurrence (**C**) and overall survival (**D**) after RFA between the two groups in the matched cohort.

We compared the differences in RFS between the ETV and TDF groups in both the full and matched cohorts. In the full cohort, the probabilities of 1-year, 3-year, and 5-year recurrence were 19.3%, 37.6%, and 41.6%, respectively, in the ETV group and 25.5%, 36.3%, and 37.3%, respectively, in the TDF group (Figure 2A). The median recurrence-free times were 46.3 (95% CI: 35.9–56.7) and 53.9 (95% CI: 8.3–99.5) months in the ETV and TDF groups, respectively. There was no significant difference between the two groups in tumor recurrence (*p* = 0.49). In the matched cohort, the probabilities of 1-year, 3-year, and 5-year recurrence were 18.5%, 36.2%, and 40.8%, respectively, in the ETV group and 28.6%, 39.0%, and 40.3%, respectively, in the TDF group (Figure 2C). The median RFS was 42.9 (95% CI: 32.6–53.2) and 53.9 (95% CI: 9.3–98.5) months in the ETV and TDF groups, respectively. There was no significant difference between the two groups in tumor recurrence (*p* = 0.35).

**Figure 3:**
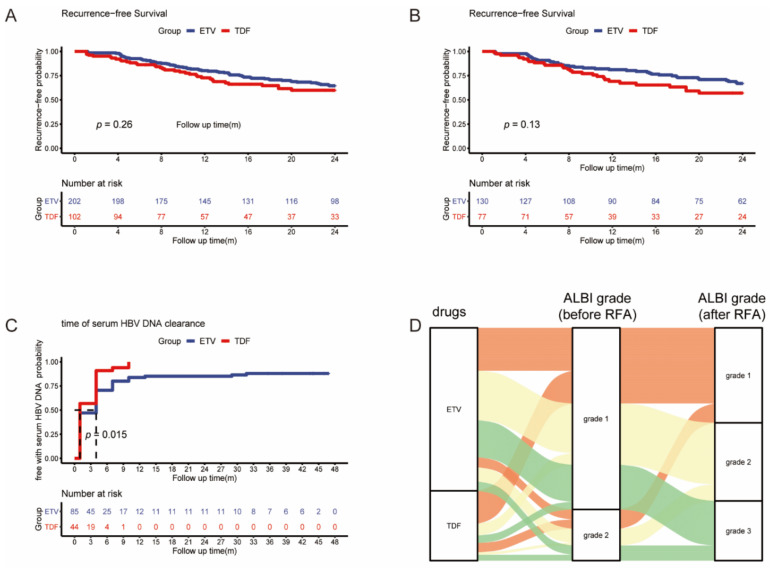
Kaplan–Meier curves of early recurrence between the two groups in full (**A**) and matched (**B**) cohorts; Kaplan–Meier curves of serum HBV DNA clearance between the two groups (**C**); Sankey diagram of ALBI grade change after RFA (**D**).

In the full cohort and matched cohort, we analyzed the early (>2 years) RFS between the ETV and TDF groups. A total of 101 patients (66 in the ETV group and 35 in the TDF group) developed early tumor recurrence. The early RFS did not differ between the two groups in the full cohort and matched cohort (*p* = 0.26, *p* = 0.13) (Figure 3A,B).

In the full cohort of 304 patients, univariable analysis revealed that larger tumor size (>2 cm) (hazard ratio (HR) = 3.65 (95% CI: 1.028–12.957), *p* = 0.045), decreased platelet count (HR = 0.990 (95% CI: 0.981–0.999), *p* = 0.038), a lower level of serum albumin (HR = 0.881 (95% CI: 0.792–0.979), *p* = 0.019), and higher ALBI grade (HR = 3.54 (95% CI: 1.283–9.77), *p* = 0.015) were significantly associated with tumor recurrence. In addition, drugs after RFA were not risk factors for tumor recurrence (HR = 1.605 (95% CI: 0.543–4.741), *p* = 0.392). Multivariable analysis revealed that larger tumor size (>2 cm) (HR = 4.089 (95% CI: 1.144–14.61), *p* = 0.03) was significantly associated with tumor recurrence (Table 3).

### 3.3. Overall Patient Survival

We compared the differences in OS between the ETV and TDF groups in both the full and matched cohorts. In the full cohort, the probabilities of 1-year, 3-year, and 5-year OS were 100%, 96.0%, and 95.0%, respectively, in the ETV group and 100%, 96.1%, and 95.1%, respectively, in the TDF group (Figure 2B). The mean duration of OS was 65.4 (95% CI: 63.6–67.2) and 62.6 (95% CI: 58.3–67.0) months in the ETV and TDF groups, respectively. There was no significant difference in OS between the two groups (*p* = 0.39). In the matched cohort, the probabilities of 1-year, 3-year, and 5-year OS were 100%, 96.9%, and 96.9%, respectively, in the ETV group and 100%, 94.8%, and 93.5%, respectively, in the TDF group (Figure 2D). The mean duration of OS was 66.5 (95% CI: 64.6–68.3) and 61.8 (95% CI: 56.8–66.7) months in the ETV and TDF groups, respectively. There was also no significant difference in OS between the two groups (*p* = 0.13).

For overall survival, univariable analysis revealed that cirrhosis (HR = 1.914 (95% CI: 1.077–3.402), *p* = 0.027), a higher level of serum ALT (HR = 1.003 (95% CI: 1–1.006), *p* = 0.023), serum HBV DAN load (HR = 1.105 (95% CI: 1.028–1.187), *p* = 0.006), and AFP level (≥20 ng/mL) (HR = 1.159 (95% CI: 1.115–2.28), *p* = 0.011) were significantly associated with unfavorable overall survival. In addition, drugs after RFA were not risk factors for overall survival (HR = 1.145 (95% CI: 0.78–1.682), *p* = 0.489). Multivariable analysis revealed that cirrhosis (HR = 1.915 (95% CI: 1.067–3.435), *p* = 0.029), a higher level of serum HBV DAN load (HR = 1.078 (95% CI: 1.003–1.159), *p* = 0.041), and AFP level (≥20 ng/mL) (HR = 1.459 (95% CI: 1.044–2.141), *p* = 0.028) were significantly associated with unfavorable overall survival (Table 3).

### 3.4. Short-Term Effect of ETV and TDF for HBV-Related Hepatitis

A total of 129 patients’ serum HBV DNA was beyond 0 UI/mL (ETV = 85, TDF = 44), and the log HBV DNA level was similar between the two groups (ETV = 4.20, TDF = 4.05, *p* = 0.614). A total of 114 patients’ serum HBV DNA was reduced to 0 (ETV = 73, TDF = 41) during the follow-up period. The mean time of serum HBV DNA being reduced to 0 was 9.13 (95% CI: 5.92–12.33) and 2.75 (95% CI: 2.01–3.49) months in ETV and TDF groups, respectively. Serum HBV DNA was reduced significantly faster in the TDF group than in the ETV group (*p* = 0.015) (Figure 3C).

One month after RFA, the serum levels of ALB and TBIL were tested in 277 patients (ETV = 194, TDF = 83), whose ALBI grades were calculated. Compared with the ALBI grade before RFA, the ALBI grade of 80 patients in the ETV group (41%) and 64 patients (64%) in the TDF group remained stable or changed to better (*p* < 0.001) (Figure 3D). TDF had a better protection effect for HBV-infected liver after RFA.

## 4. Discussion

In the present study, we analyzed 304 HBV-related HCC patients who were treated with ETV (*n* = 202) or TDF (*n* = 102) after RFA. We found that TDF therapy was associated with a better effect of protecting liver function and reducing HBV DNA loads compared with ETV therapy, but there was no difference in recurrence and overall survival, which was consistently observed in full and propensity-score-matched cohorts.

It has been acknowledged that patients are recommended to take NAs, which can reduce the risks of recurrence and death after curative treatment of HBV-related HCC including hepatic resection and RFA [14,32,33,34,35]. However, ETV and TDF are equally recommended by international practice guidelines as first-line antiviral agents for CHB among NAs. Consequently, many researchers focused on comparing the effect of ETV and TDF to guide which was more suitable for HBV-related HCC patients.

For the short-term effect, a previous meta-analysis including 11 studies with 1656 patients reported that TDF was better able to suppress HBV viral load in treating chronic HBV patients [36]. Virologic response (VR), which was defined as a serum HBV DNA level less than 60 IU/mL at 1 year of treatment, was used to evaluate the ability to suppress HBV viral load. Missing or unavailable VR data were considered as a failure of VR. In our study, we recorded the change in serum HBV DNA and ALBI grade after RFA, and we found that serum HBV DNA was reduced significantly faster in the TDF group than in the ETV group and TDF had a better protection effect for HBV-infected liver after RFA. In comparison with previous studies, we used Kaplan–Meier curves to estimate the time of serum HBV DNA clearance of the two patient groups and compared curves by the log-rank test. Because of the high antiviral effects of ETV and TDF, serum HBV DNA of most patients was reduced to 0, so we defined serum HBV DNA being reduced to 0 as the event for the time of serum HBV DNA clearance. The follow-up duration was defined as the interval between the first RFA and either the incidence of event or the last follow-up time. That missing or unavailable VR data were considered as a failure of VR may cause bias in previous studies. However, Kaplan–Meier curves can reduce the bias even though VR data of some patients were missing or unavailable. Therefore, the time of serum HBV DNA clearance may be more accurate to evaluate the ability to suppress HBV viral load.

For the long-term effect, whether ETV or TDF therapy results in a better prognosis remains controversial. For patients with CHB, some studies showed that TDF was associated with a low risk of HCC incidence compared with ETV [37,38], but Kim et al. [39] and Hsu et al. [40] reported that HCC incidence was not significantly different between the ETV and TDF groups. For patients with HBV-related HCC, two recent studies showed that TDF treatment was associated with a significantly lower rate of HCC recurrence and better overall patient survival than ETV treatment among patients after curative hepatectomy for HBV-related HCC [26,41]. However, Lee et al. failed to confirm such outcomes. Their results showed no different outcomes between the two groups [27].

However, there are few studies about the effect of ETV vs. TDF on the outcomes of HBV-related HCC patients after curative RFA. Only the subgroup analysis of Lee showed the recurrence and overall survival were similar in patients after RFA between ETV and TDF groups [27]. The conclusion of our study is consistent with Lee’s. In this study, we investigated the outcomes of recurrence and overall patient survival in these two groups. In the univariable and multivariable analysis, it was shown that several baseline factors were associated with tumor recurrence and overall survival after RFA: serum PLT, ALT, HBV DNA, AFP, ALBI grade, tumor size, and the presence of cirrhosis in the full cohort. To minimize the effect of risk factors other than antiviral treatment between the two groups, we analyzed the data using propensity score matching. After propensity score matching, there was no significant difference in risk factors between these two groups. Our study showed that the tumor recurrence and overall survival were not significantly different between these two groups in the full cohort and the matched cohort. The early recurrence was not significantly different in the full cohort and the matched cohort either.

In summary, we found that TDF was better in short-term effects, but there was no difference in long-term effects. It is unclear why the short-term effects do not extend to long-term effects. We thought that in spite of TDF having a better ability to suppress HBV viral load, both ETV and TDF have high antiviral effects and a high genetic barrier to drug resistance. Within 1 year, the viral load of 293 patients (96.4%) was at the level of 0. The viral loads of most patients, whether in the ETV group or the TDF group, were in good control, which led to no difference in long-term effect.

Our study has several strengths. First, we excluded incomplete ablation (1.9%) and LTP patients (4.0%) to eliminate the effect of technical RFA failure, which makes the conclusion more convincing. Second, compared to Lee’s study [27], we limited our study population to patients who received RFA as the initial therapy for HCC to eliminate the effect of confounding factors. Third, we estimated the curative effect of ETV and TDF for HBV-related hepatitis by recording the change in serum HBV DNA and ALBI grade after RFA.

Our study has several limitations. First, any single-center retrospective study is associated with a risk of selection bias. A randomized controlled trial can provide more convincing conclusions, so we are currently conducting a prospective randomized study that compares the effects of ETV and TDF on outcomes of patients after curative therapy for HBV-related HCC. Second, the starting time point of oral antiviral treatment after RFA was not consistent because this study was conducted as a retrospective study. The duration of antiviral treatment may affect the outcomes of patients. Third, our results may not be generalizable to all HBV-related HCC patients who underwent curative therapy because we limited the population to patients who received RFA. Last, the biological aggressiveness of the tumor was lacking in baseline characteristics because histologic features cannot be obtained in terms of RFA therapy, unlike in cases of surgical resection.

## 5. Conclusions

In conclusion, the RFS and OS of patients who underwent curative RFA for HCC were not significantly different between ETV and TDF groups. TDF therapy was associated with a better effect of protecting liver function and reducing HBV DNA loads. Further validation studies are needed.

## Figures and Tables

**Table 1:** Baseline characteristics of all patients (*n* = 304).

	ETV Group	TDF Group	*p*-Value
	(*n* = 202)	(*n* = 102)	
Age (years)	53 (46, 63)	53.5 (46, 61.8)	0.592
Gender			0.054
women	31 (15)	7 (7)	
men	171 (85)	95 (93)	
Diabetes (*N*, %)			0.378
absence	185 (92)	97 (95)	
presence	17 (8)	5 (5)	
Hypertension (*N*, %)			0.455
absence	178 (88)	86 (84)	
presence	24 (12)	16 (16)	
Complications (*N*, %)			0.004
absence	180 (89)	101 (99)	
presence	22 (11)	1 (1)	
Cirrhosis (*N*, %)			0.008
absence	27 (13)	27 (26)	
presence	175 (87)	75 (74)	
Tumor size (cm)	2.2 (1.7, 2.8)	2.3 (1.7, 2.8)	0.653
Tumor number (*N*, %)			0.152
1	190 (94)	100 (98)	
2	12 (6)	2 (2)	
Platelet (×10^3^/mm^3^)	148.5 (107, 192)	169.5 (122.3, 200)	0.060
PT (s)	12.2 (11.6, 13)	12 (11.6, 12.7)	0.153
APTT (s)	28.4 (25.9, 31.2)	27.2 (25.2, 29.6)	0.019
Albumin (g/dL)	43.1 (40.4, 45.4)	43.8 (40.4, 46.7)	0.194
Total bilirubin (mg/dL)	14.3 (10.6, 20.1)	13.4 (10.8, 18.1)	0.563
ALT (U/L)	31.7 (23.1, 45.5)	39.15 (25.1, 52.4)	0.014
AST (U/L)	29.7 (23.6, 38.3)	34.1 (26.1, 42.5)	0.015
Log_10_HBV DNA (IU/mL)	2.1(1.1, 2.3)	2.4 (1.5, 2.7)	0.199
AFP (ng/mL)	16.66 (4.14, 158.88)	12.82 (4.72, 132.78)	0.857
AFP (ng/mL)			1
<20	107(53)	54 (53)	
≥20	95 (46)	47 (46)	
Child–Pugh class (*N*, %)			0.305
A	198 (98)	102 (100)	
B	4 (2)	0 (0)	
ALBI (*N*, %)			1
Grade 1	159 (79)	80 (78)	
Grade 2	43 (21)	22 (22)	

Categorical variables are described as frequencies and percentages. Continuous variables are described as mean ± standard deviation (SD) and median with interquartile range for parametric and nonparametric variables, respectively.

**Table 2:** Baseline characteristics of patients (*n* = 207) after PSM.

	ETV Group	TDF Group	*p*-Value
	(*n* = 130)	(*n* = 77)	
Age (years)	53 (46.25, 64)	54 (46, 62)	0.856
Gender			0.276
women	18 (14)	6 (8)	
men	112 (86)	71 (92)	
Diabetes (*N*, %)			0.434
absence	118 (91)	73 (95)	
presence	12 (9)	4 (5)	
Hypertension (*N*, %)			0.302
absence	113 (87)	62 (81)	
presence	17 (13)	15 (19)	
Complications (*N*, %)			1
absence	130 (100)	77 (100)	
Cirrhosis (*N*, %)			1
absence	20 (15)	12 (16)	
presence	175 (87)	75 (74)	
Tumor size (cm)	2.2 (1.7, 2.8)	2.2 (1.7, 2.6)	0.819
Tumor number (*N*, %)			0.095
1	121 (93)	76 (99)	
2	9 (7)	1 (1)	
Platelet (×10^3^/mm^3^)	150 (108.5, 195)	157 (112, 195)	0.952
PT (s)	12.1 (11.53, 12.7)	12.1 (11.6, 12.7)	0.708
APTT (s)	28.1 (25.67, 30.63)	27.6 (25.4, 30.2)	0.477
Albumin (g/dL)	42.96 ± 3.67	43.73 ± 4.34	0.171
Total bilirubin (mg/dL)	14.85 (10.7, 20.07)	13.3 (11.1, 18.1)	0.342
ALT (U/L)	32.5 (22.22, 42.08)	34.2 (21.5, 44.2)	0.5
AST (U/L)	28.75 (23.23, 37.15)	32.7 (24.7, 39)	0.199
Log_10_HBV DNA (IU/mL)	0 (0, 4.22)	2.08 (0, 4.7)	0.161
AFP (ng/mL)	13.37 (3.98, 158.88)	15.52 (4.85, 120.1)	0.884
AFP (ng/mL)			0.533
<20	73 (56)	39 (51)	
≥20	57 (44)	38 (49)	
Child–Pugh class (*N*, %)			0.305
A	198 (98)	102 (100)	
B	4 (2)	0 (0)	
ALBI (*N*, %)			0.715
Grade 1	107 (82)	61 (79)	
Grade 2	23 (18)	16 (21)	

Categorical variables are described as frequencies and percentages. Continuous variables are described as mean ± standard deviation (SD) and median with interquartile range for parametric and nonparametric variables, respectively.

**Table 3:** Univariate and multivariate analysis of risk factors for overall survival and recurrence-free survival.

Variables	Overall Survival						Recurrence-Free Survival					
	Univariate Analysis			Multivariate Analysis			Univariate Analysis			Multivariate Analysis		
	HR	95% CI	*p*	HR	95% CI	*p*	HR	95% CI	*p*	HR	95% CI	*p*
Age (years)	1.019	0.971–1.069	0.448				0.997	0.981–1.013	0.7			
Gender (man/woman)	0.720	0.202–2.56	0.661				0.699	0.44–1.11	0.129			
Diabetes (yes/no)	1.745–7.755	0.393–7.755	0.464				1.198	0.645–2.227	0.568			
Hypertension (yes/no)	1.643	0.461–5.849	0.444				0.820	0.477–1.408	0.472			
Complications (yes/no)	0.933	0.204–4.257	0.929				0.931	0.497–1.741	0.822			
Cirrhosis (yes/no)	1.149	0.259–5.105	0.855				1.914	1.077–3.402	0.027	1.915	1.067–3.435	0.029
Tumor size (≥2 cm/<2 cm)	3.65	1.028–12.957	0.045	4.089	1.144–14.61	0.03	1.083	0.756–1.551	0.664			
Tumor number (1/2)	0	0–∞	0.998				1.076	0.473–2.445	0.861			
Platelet (×10^3^/mm^3^)	0.990	0.981–0.999	0.038	0.992	0.982–1.002	0.111	0.998	0.996–1.001	0.27			
PT (s)	1.334	0.916–1.941	0.133				0.919	0.767–1.101	0.361			
APTT (s)	1.06	0.957–1.173	0.264				0.973	0.933–1.015	0.204			
Albumin (g/dL)	0.881	0.792–0.979	0.019				0.973	0.933–1.014	0.195			
Total bilirubin (mg/dL)	1.102	0.951–1.076	0.709				0.978	0.952–1.004	0.092			
ALT (U/L)	1.004	0.993–1.015	0.551				1.003	1–1.006	0.023	1.003	1–1.007	0.059
AST (U/L)	1.005	0.994–1.015	0.391				1.002	0.999–1.006	0.145			
Log_10_HBV DNA (IU/mL)	0.870	0.685–1.106	0.256				1.105	1.028–1.187	0.006	1.078	1.003–1.159	0.041
AFP (≥20 ng/mL/<20 ng/mL)	3.016	0.959–9.485	0.059				1.159	1.115–2.28	0.011	1.459	1.044–2.141	0.028
Child–Pugh class (B/A)	0	0–∞	0.998				0.496	0.069–3.553	0.485			
ALBI grade (2/1)	3.54	1.283–9.77	0.015	2.364	0.782–7.149	0.128	1.301	0.862–1.966	0.211			
Drugs after RFA (ETV/TDF)	1.605	0.543–4.742	0.392				1.145	0.78–1.682	0.489			

HR: hazard rate; CI: confidence interval; HBV: hepatitis B virus; PLT: blood platelet; ALT: alanine aminotransferase; AST: aspartate aminotransferase; AFP: alpha-fetoprotein; PT: prothrombin time; APTT: activated partial thromboplastin time; ALBI grade: albumin–bilirubin grade.

## Data Availability

All data generated or analyzed during this study are included in this article. Further inquiries can be directed to the corresponding author.
